# Conformational plasticity and evolutionary analysis of the myotilin tandem Ig domains

**DOI:** 10.1038/s41598-017-03323-6

**Published:** 2017-06-21

**Authors:** Vid Puž, Miha Pavšič, Brigita Lenarčič, Kristina Djinović-Carugo

**Affiliations:** 1 Department of Biochemistry, Faculty of Chemistry and Chemical Technology, University of Ljubljana, Večna pot 113, SI-1000 Ljubljana, Slovenia; 2 Department of Biochemistry, Molecular Biology and Structural Biology, Jožef Stefan Institute, Jamova 39, SI-1000 Ljubljana, Slovenia; 3 Department of Structural and Computational Biology, Max F. Perutz Laboratories (MFPL), University of Vienna, Campus Vienna Biocenter 5, A-1030 Vienna, Austria

## Abstract

Myotilin is a component of the sarcomere where it plays an important role in organisation and maintenance of Z-disk integrity. This involves direct binding to F-actin and filamin C, a function mediated by its Ig domain pair. While the structures of these two individual domains are known, information about their relative orientation and flexibility remains limited. We set on to characterise the Ig domain pair of myotilin with emphasis on its molecular structure, dynamics and phylogeny. First, sequence conservation analysis of myotilin shed light on the molecular basis of myotilinopathies and revealed several motifs in Ig domains found also in I-band proteins. In particular, a highly conserved Glu344 mapping to Ig domain linker, was identified as a critical component of the inter-domain hinge mechanism. Next, SAXS and molecular dynamics revealed that Ig domain pair exists as a multi-conformation species with dynamic exchange between extended and compact orientations. Mutation of AKE motif to AAA further confirmed its impact on inter-domain flexibility. We hypothesise that the conformational plasticity of the Ig domain pair in its unbound form is part of the binding partner recognition mechanism.

## Introduction

Movement is vital to all living organisms, from the transport of single molecules in cells to the movement of the entire organism. In vertebrates, skeletal muscle is essential for all voluntary movements such as walking, running, swimming or flying. Involuntary movements, such as those occurring in cardiac muscle resulting in contraction of the heart (beating), or those occurring in smooth muscle resulting in peristalsis are fundamental to the viability of the organism.

Muscles are composed of repetitive isotropic (I) and anisotropic (A) bands, giving them a striped appearance. Here, the smallest periodic unit, termed sarcomere, is composed of actin and myosin filaments which together with other accessory proteins form the sarcomeric cytoskeleton^1, 2^. The precise ultra-structural order of these filaments is of outmost importance for converting the molecular interactions produced by actin and myosin in each sarcomere into efficient contraction at the macroscopic level. Titin and nebulin (and its smaller cardiac variant nebulette) act as a general blueprint for sarcomere assembly and length, due to their outstanding size and modular architecture. Sarcomere is on both ends delimited with Z-discs, which act as passive transducers of the generated tension. This is also the spot of actin filament crosslinking, primarily by the α-actinin, however other signalling, stretch-sensing and actin-binding proteins are involved, for example myotilin, filamin C, ZASP/cypher/oracle, FATZ/myozenin/calsarcin and many others^3^.

**Figure 1:**
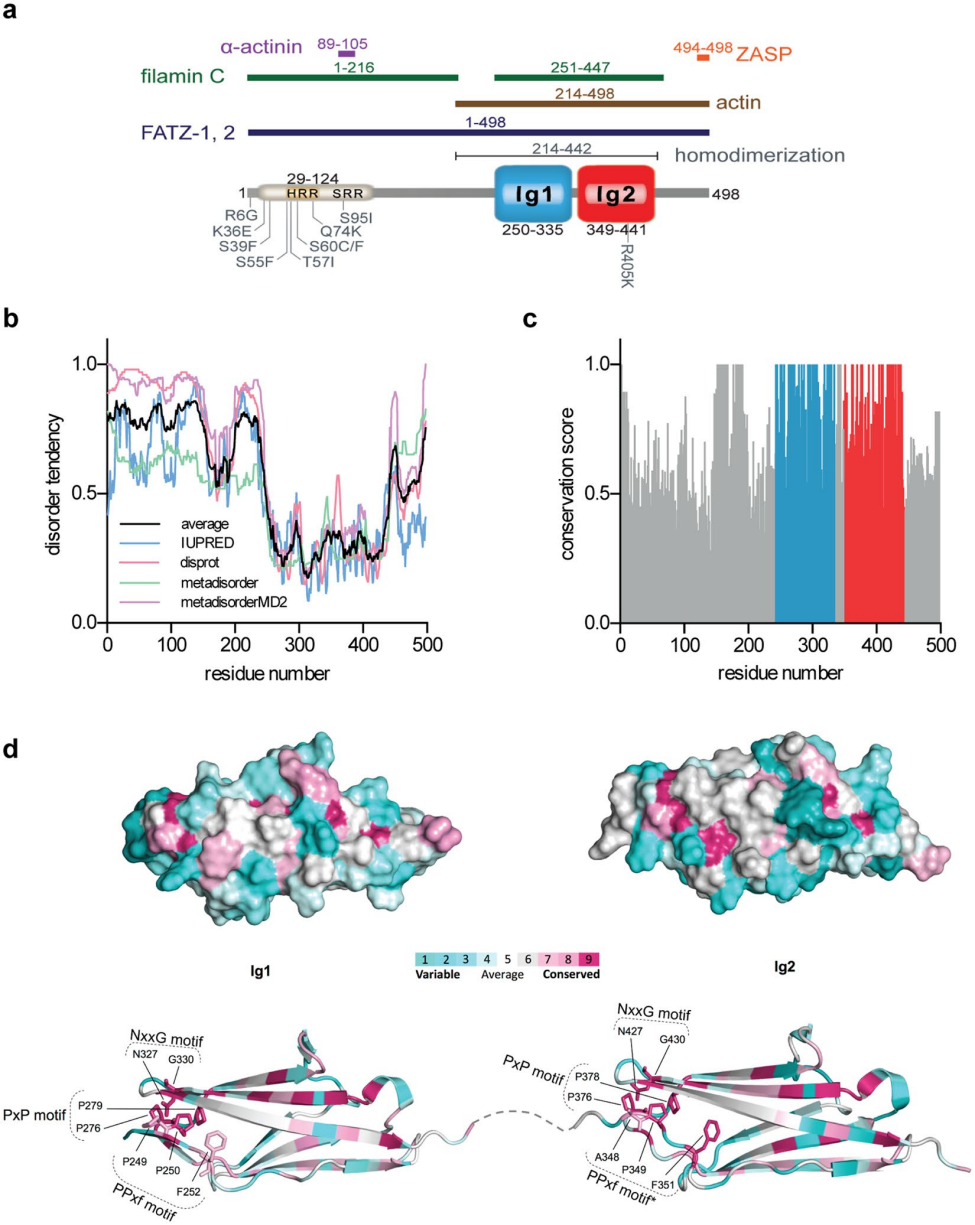
Evolutionary analysis of myotilin. (**a**) Schematic presentation of the myotilin molecule. N-terminal part of myotilin is indicated by the serine-rich region, comprised of »mutational hotspot« and hydrophobic residues stretch (yellow). C-terminal Ig domains 1 and 2 are coloured blue and red, respectively, followed by the C-terminal tail. Binding sites for various binding partners are noted above schematics. (**b**) Disorder tendency plot, calculated with the different prediction software, showing that mainly Ig domains present a structured part of the molecule. (**c**) Conservation plot of every amino acid residue score, calculated from the multiple-sequence alignment. (**d**) Conservation of the Ig domains of myotilin as calculated by the Consurf-DB server. Below are presented conserved residues, forming an N-terminal cluster. The colouring scheme presents purple residues as conserved and turquoise as varied. *PPxf motif on Ig2 domain is APxf, whereas sequences from other organisms possess typical PPxf motif (Supplementary Data S1).

Myotilin, the founding member of the myotilin/myopalladin/palladin family was initially identified and characterised as α-actinin binding protein^4^. Both myopalladin and myotilin have restricted expression profile, with the highest expression in the skeletal and cardiac muscle^4, 5^, in contrast to palladin, which shows much wider expression pattern both in skeletal and non-skeletal tissues, depending on the expressed isoform^6, 7^. Myotilin is comprised of two C2-type Ig domains and a unique serine-rich N-terminal region^4^, while palladin and myopalladin host up to five Ig domains, where the two most C-terminal domains share high homology with the Ig domains of myotilin and two most N-terminal domains are mostly related to the N2B region of titin^5^ (Fig. 1a). Ig-like domains are required for the myotilin dimerisation^4, 8^ and interaction with other binding partners such as G- and F-actin^8, 9^, filamin C^10^ and FATZ^11^. With the motif ^494^ESEEL^498^ at the very C-terminal tail of the molecule myotilin is able to bind to the PDZ domain of protein ZASP, similarly to the C-terminal motif of α-actinin and FATZ family of proteins^12^. At the N-terminal part, encompassing residues 89–105, myotilin binds to the calmodulin-like domain of α-actinin-2 (ref. 13). More specifically, via canonical motif 1-4-5-8 (numbers correspond to sequence positions of hydrophobic amino acid residues) myotilin is able to interact with EF3-4 hands of α-actinin, in a similar way as it was shown for titin Z-repeats^14–17^, palladin^13, 18^ and more recently for neck peptide of α-actinin-2 in its auto-inhibited form^19^.

Myotilin is thought to play a role in anchoring and stabilising actin filaments at the Z-disc, and is involved in the organisation and maintenance of Z-disk integrity^12^. Its involvement in actin crosslinking, bundling in concert with α-actinin-2 and its interactions with important components of Z-disc, suggest that it plays a role in structural stabilisation and assembly of the Z-disc^20^. During the early stages of myofibrillogenesis, myotilin is present in the pre-myofibrills (Z-bodies) together with actin, α-actinin-2, filamin C, FATZ and ZASP^21^. Recent results reported that it is highly mobile not only in the Z-bodies but also in the Z-bands and possesses ability of dynamic exchange with the cytoplasmic pool of proteins, indicating important role in initial organisation and maintenance of Z-disc^22^.

The N-terminal part of molecule, which is predicted to be intrinsically unstructured (Fig. 1b) also represents a »mutational hotspot«, where missense mutations (all but two residing between residues 29 and 124) have been shown to cause muscle disorders such as limb-girdle muscular dystrophy 1A, myofibrillar myopathy, and spheroid body myopathy^20, 23–26^ (Fig. 1a), collectively termed myotilinopathies^24, 27^. Missense disease mutations in myotilin were shown to have no impact on the localisation and dynamics in the Z-disc, however degradation rates were slower for the mutant forms of myotilin, compared to the wild-type^28^. It is important to note that members of the family may have overlapping functions, such as binding to the same partner and can probably compensate for each other, as it was shown with the *myo* ^−/−^ mice, which did not show any abnormalities or differences, compared to the normal mice^29^. Although several properties of myotilin as described above propose its role in the Z-disc, its primary function still remains unknown.

It is well established that function of several sarcomeric proteins critically depends on the flexibility/rigidity of their tandem Ig domain repeats. For example, flexibility of tandem Ig repeats in filamin directly affects its interactions with other proteins^30, 31^. In titin, inter-domain flexibility is essential for its mechanical functions and stability^32–38^. More specifically, titin Z1Z2 tandem domains, localized at the Z-disc, possess an adaptable dynamics for recruitment of the binding partner telethonin^34^. However, no such data exists for the myotilin tandem Ig pair. Since myotilin is a member of tandem Ig domain containing proteins, homologous to titin, it can be expected that the inter-domain flexibility could have an impact on its interaction with the diverse proteins and, in turn, the mechanical properties of the sarcomeric cytoskeleton. To gain insight into its dynamic properties we used several complementary computational and experimental approaches. First, we applied molecular evolution approach to analyse myotilin conservation across homologous sequences with the aim to identify region of flexibility. Next, we examined structural properties and dynamics of myotilin Ig domains in solution by using SAXS measurements and molecular dynamics simulations.

## Results

### Evolutionary analysis of myotilin

Molecular evolution analysis has already proved to be a valuable tool for identification of evolutionary relationship between Ig domains of different sarcomeric proteins^39–41^. To gain insight into myotilin amino acid sequence conservation we assembled a dataset of 85 homologous sequences of Vertebrates subphyla ranging from Chondrichthyes to Mammalia classes and constructed a multiple sequence alignment. Myotilin homologues of the evolutionarily distant species of the Chordata phylum could not be identified; here, the highest sequence similarity was observed for Ig domains of titin and titin-like proteins, which were not included in the analysis. For comparison, a sequence alignment using only one representative species of each class (i.e. excluding redundant sequences from Mammalia and Aves classes) was constructed and showed similar results. The Bio-NJ tree calculated using maximum likelihood approach showed the expected evolutionary relationship between species with a good branch support for main branches indicating a reliable sequence alignment (Supplementary Fig. S1). Interestingly, detailed inspection of the alignment showed insertion of 11 residues at the N-terminal part in the sequences from the Aves class which was not observed in the other analysed sequences (Supplementary Data S1).

Per residue conservation scores clearly demonstrate that Ig domains 1 and 2, together with the short region (residues 160–190), represent the most conserved part of the molecule (Fig. 1c), which is in line with their functional role in dimerisation and interactions with other proteins. As expected, mapping of conservation score to the Ig domains’ structure revealed the highest conservation for residues of the hydrophobic core, specifically the tryptophan residues in the centre of each of the domains (W283 in Ig1 and W382 in Ig2), which are critical for the stability of the immunoglobulin fold and form together with other hydrophobic residues a folding nucleus^42^. On the other hand, surface residues are less conserved with the exception of three important motifs: PPxf, NxxG and PxP. The PPxf motif (x = any residue, f = a hydrophobic residue, most often Phe) is located at the N-terminus of both Ig domains and has already been found in Ig domains of other proteins from the I-band^43^. Interestingly, conserved proline residues, first identified at the border of fibronectin type III domains of tenascin, were shown to prevent non-specific self-association and aggregation of their cognate proteins^44^. We speculate that similar effects could be also present in myotilin. The NxxG motif (x = any residue) present at the β-hairpin turn connecting the F and G strands, is a central part of the N-terminal network of conserved residues. Similarly to the PPxf motif, it is found in the N-conserved type of I-band Ig domains^33^. Together with the PxP motif, located at the BC loop on both domains, these motifs form a conserved N-terminal network of residues (Fig. 1d). Overall, these results demonstrate that throughout the course of evolution certain structurally and/or functionally important features of Ig domains were retained while other parts, particularly solvent-exposed regions, are more varied to accommodate different binding partners.

Next, inspection of alignment of the linker region within tandem Ig domain repeat of myotilin sequence (^341^AKEHKR^346^) and corresponding repeats in myopalladin (^1165^AKEVKK^1170^) and palladin (^1227^AKEAHK^1232^) revealed that Ala, Lys and Glu residues are highly conserved within this region with an overall basic character. Interestingly, highly conserved Glu is also characteristic for I-band linker regions where it sometimes merges with PPxf to form the EPPxf motif. Mutational studies of this motif within the titin Ig65–70 domain repeats suggested that it is not a determinant of inter-domain orientation^33^, however latest studies of tandem titin Ig67–68 repeats indicate that it is important for titin global dynamics^38^. While myotilin lacks the combined EPPxf motif, the PPxf motif could nevertheless be involved in inter-domain dynamics. Even more, similar inter-domain dynamics are expected for domain pairs Ig1–2 (myotilin) and Ig4-Ig5 (myopalladin/palladin) due to their high sequence similarity and linker length. Linker regions within other Ig domain pairs of myopalladin/palladin are longer (76 and 44 residues for Ig1–2 and Ig3–4, respectively) indicating a higher degree of flexibility.

In contrast to the two Ig domains of myotilin, the N-terminal (residues 1–250) and the C-terminal part (residues 441–498) display lower conservation scores. While those two regions contain functionally important sites (binding sites for α-actinin-2 on N-terminus, and ZASP binding site at the very C-terminus (Fig. 1a), detailed structural analysis indicates that they are intrinsically disordered (Fig. 1b). Link between intrinsic disorder and higher sequence variation is in line with the observation that disordered regions typically evolve faster (i.e., have higher mutation rate) than the structured domains^45^. This was also observed for myopalladin and palladin (Supplementary Fig. S1) where unstructured parts are mainly located between the Ig domains. Still, the structure of such regions could be induced/stabilised upon binding of another protein. A representative example is the formation of an α-helical structure at the otherwise unstructured N-terminus of palladin upon binding to EF34 hands of α-actinin-2 (ref. 13).

### Analysis of disease-causing mutations in myotilin

Several mutations within the myotilin gene, mostly mapping to the intrinsically disordered N-terminal part, result in Z-disc alterations and polymorphous aggregate formation, which is associated with different myopathies^20, 23–26^. Therefore, we performed detailed inspection of the disease-linked mutations to gain an insight into their possible common denominator. Conservation scores of the mutation hotspots showed no significant differences compared to the other regions; average conservation score for all mutations was 0.673, where 0 present fully variable and 1 fully conserved score. Majority of the mutations are substitutions from polar/charged residues to a hydrophobic residue with the exception of K36E, Q74K and R405K (Fig. 1a). The newly formed hydrophobic clusters could together with the nearby hydrophobic rich region (HRR, Fig. 1a) of the molecule promote self-association and formation of aggregates. Since almost all mutations are located within the intrinsically disordered region we speculate that mutations could positively affect disorder-to-order transition and thereby enabling further progression of aggregation. To test this hypothesis, we compared the disorder tendency plots for wt myotilin and the disease-related mutants (Supplementary Fig. S2). Mutations resulting in introduction of hydrophobic residues displayed reduced disorder while other mutations (K36E, Q74K and R405K) did not show significant differences. Similar effect of mutations on the disorder-to-order transition was also predicted for the large dataset of disordered proteins, and more specifically for the mutation R243W of the disordered region of p63 (ref. 46). Moreover, in γC-crystallin the mutation R168W, directly linked to congenital cataracts, increased aggregation and precipitation propensity, possibly due to the increased exposure of a hydrophobic patch^47^.

### Ig domain pair displays conformational plasticity

#### SAXS analysis of tandem Ig domains

To examine structural properties of the tandem Ig domains of myotilin in solution, small-angle X-ray scattering (SAXS) was employed. SAXS data of MYOTIg1–2 construct encompassing the two Ig domains was measured using highly purified samples of myotilin Ig domains at different concentrations. Slightly increased radius of gyration (R_g_) at the highest concentration indicates some concentration effect, however this did not obstruct further analysis (Supplementary Fig. S3). It is well established that high protein concentrations may lead to inter-particle repulsion or attraction, which influences the scattering and in turn affects interpretation of the results^48^. Therefore, the scattering curve was extrapolated to zero concentration to minimise the effect of inter-particle attraction without obstructing good signal-to-noise ratio. Calculation of molecular weight clearly showed that MYOTIg1–2 construct was monomeric under used experimental conditions.

**Figure 2:**
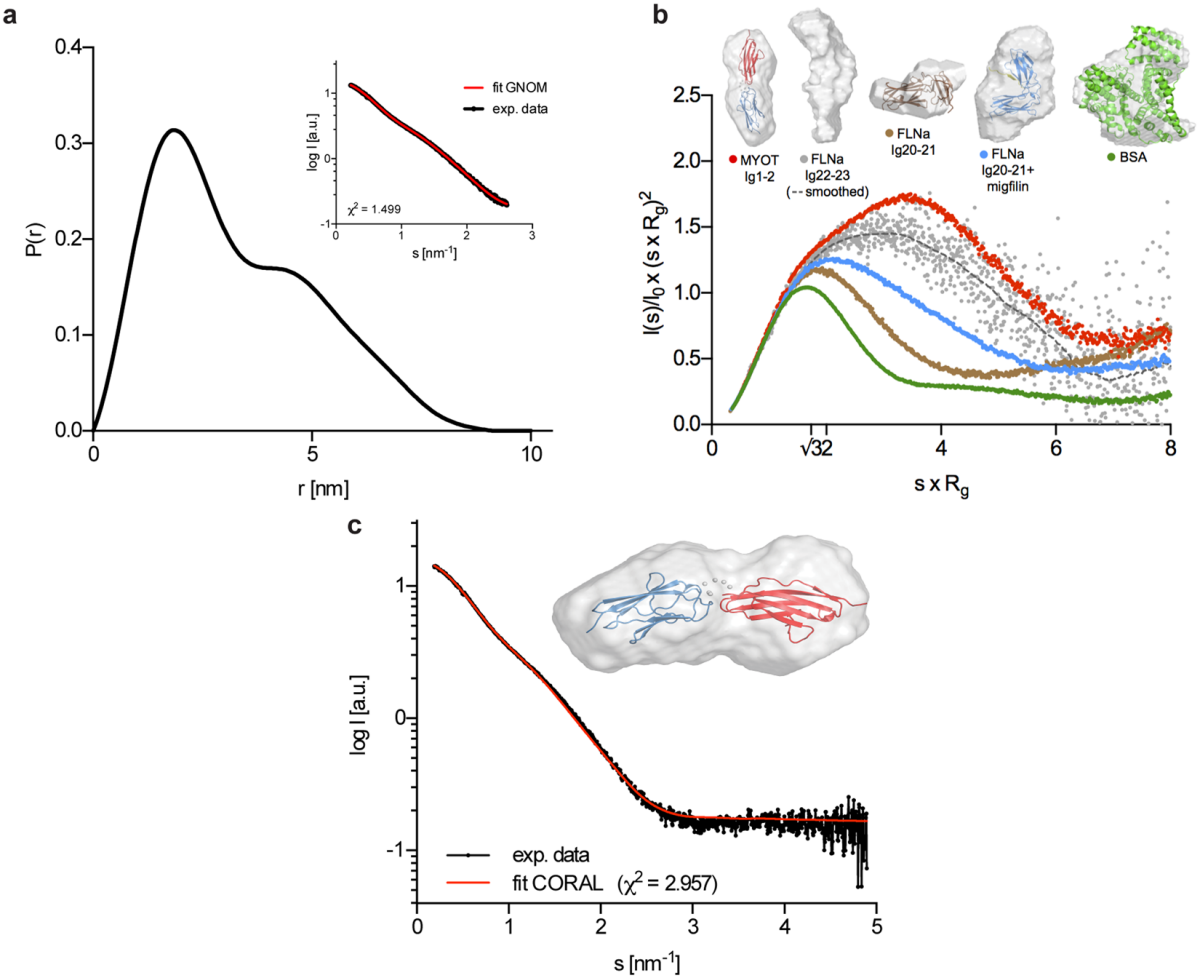
SAXS analysis of myotilin Ig domains. (**a**) P(r) *vs*. r plot for MYOTIg1-2, showing bimodal profile, with the D_max_ = 9.37 nm. Curve is showing smooth decay to P(r)=0 at the D_max_ and good fit to the experimental data presented in the inset, using GNOM program (s = 4πsinθ/λ). (**b**) Comparison of dimensionless Kratky plots of MYOTIg1-2 with different proteins as an examples of globular protein (BSA), tandem Ig domains of filamin A with compact conformation (Ig20-21), and the same construct with the addition of migfilin peptide (Ig20-21+migfilin), which increases flexibility of the Ig domains. MYOTIg1-2 shows less compact conformation with the degree of flexibility as indicated by Kratky plot broadening and maxima shift and is similar to Ig22-23 of filamin A. Molecular envelopes of the analysed proteins, with the available high-resolution structures are presented above graph. (**c**) Fit of the calculated rigid body model by CORAL to experimental scattering with goodness of the fit expressed in χ^2^. Superposition of the rigid body model with the averaged *ab initio* reconstructed molecular envelope of MYOTIg1-2 is presented in the inset.

Guinier region analysis of the scattering curve showed linear fit (Supplementary Fig. S3) with the values I_0_ = 15.34 ± 0.06 a.u. and R_g_ = 2.57 ± 0.10 nm. Pair-density distribution function P(r), displays a bell-shaped bimodal profile, typical for a multi-domain protein. The fit of P(r) to experimental data shows good match and smooth decay to P(r) = 0 and represents particle with maximum linear dimension D_max_ = 9.37 nm (Fig. 2a). Calculated values of I_0_ and R_g_ from P(r) are in excellent agreement with the values determined by Guinier approximation. Other parameters derived from scattering data are given in Table 1. D_max_ and R_g_ values of MYOTIg1–2 are between those of a fully extended Ig8–9 pair (D_max_ = 10.10 nm) and semi-extended Ig6–7 pair of filamin C (D_max_ = 8.60 nm)^49^, suggesting that the MYOTIg1–2 pair adopts a slightly more compact form than the fully extended Ig8–9.

**Table 1:** Parameters derived from the SAXS data for MYOTIg1-2.

Name	I_0_ (Guinier)[a.u.]	I_0_ (P(r))[a.u.]	R_g_ (Guinier)[nm]	R_g_ (P(r))[nm]	D_max_ [nm]	V (Porod)[nm^3^]	M_r_ (calc.)[kDa]	M_r_ (I_0_)[kDa]	M_r_ (Porod)[kDa]
MYOT Ig1-2	15.34 ± 0.06	15.37	2.57 ± 0.10	2.63	9.37	29.14	22.3	16.3	16.4

Information from the dimensionless Kratky plot suggested that on average myotilin Ig domain pair does not adopt a single conformation but instead displays a high degree of flexibility. For example, compared to bovine serum albumin (BSA)^50^, a compact globular protein, myotilin shows a less globular shape which is indicated by Kratky plot curve broadening and the maxima shifted to higher s × R_g_ values. Even more, when compared to the Ig domain pair (Ig20–21) of filamin A^31^, both in its compact and slightly more extended/flexible migfilin peptide-bound form, myotilin Ig domain pair is significantly more flexible, similarly as Ig domain pairs Ig12–13 and Ig22–23 of filamin A^51^ (Fig. 2b). Here, increased flexibility is correlated to the extended orientation of the tandem Ig domains with less frequent/stable inter-domain contacts. Therefore, in MYOTIg1–2 being the most flexible of the shown examples, the extended SAXS envelope corresponds to a nearly coaxial domain orientation on average where the two Ig domains do not form stable contacts.

To gain detailed insight into the relative orientation and dynamics of the two domains of the Ig1–2 pair, both rigid body and flexible/ensemble fitting approaches were used and assessed with regard to χ^2^ and inter-domain clashes. For rigid body fitting, the *ab initio* three-dimensional low-resolution molecular envelope was reconstructed using DAMMIF. Next, the most probable averaged model was calculated with the DAMAVER set of programs, showing a »dumbbell« shape with dimensions of approximately 10 × 4.5 nm. Subsequently, rigid body modelling using CORAL was performed, showing relatively good chi value (χ^2^ = 2.957), however with a slight mismatch at the higher angles (Fig. 2c). The superposition of rigid body model with the *ab initio* envelope suggests a rather extended conformation of the domains (Fig. 2c, inset).

Based on the Kratky plot analysis, we further explored flexibility of the tandem Ig domains using ensemble fitting. For ensemble fitting, ensemble optimisation method (EOM) and MultiFoXS approaches were employed to fit the theoretical scattering intensities (calculated from a pool of models with different inter-domain orientations) to the experimental scattering data. EOM results indicate various inter-domain orientations which are represented by four models ranging from more compact to a more extended arrangement, fitting the experimental scattering with a low chi value (χ^2^ = 1.481). Radius of gyration distribution plot shows three peaks with maxima ranging from 2.3 nm (compact), 2.7 nm (average extended), and 3.0 nm representing fully extended species (Supplementary Fig. S4). Similarly, end-to-end distance and D_max_ distribution plots show bimodal profiles, thereby additionally confirming flexibility of the Ig1–2 system.

**Figure 3:**
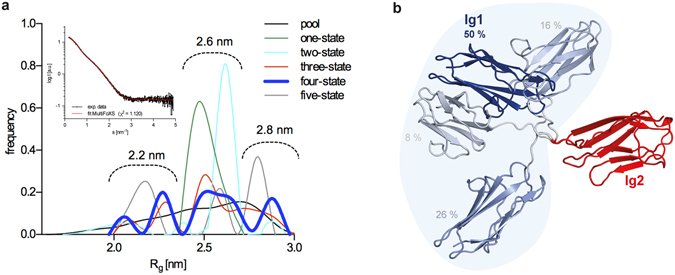
Flexibility assessment of the tandem Ig domains. (**a**) R_g_ distribution plot among 100 best scoring N-state models (N = 1, 2, 3, 4, 5), where 4-state model, that best match the experimental data, is shown in bold. Convergence of the peaks from N-state models, showing three peaks with the maxima at approximately 2.2, 2.6 and 2.8 nm. Overlay of the experimental scattering data with the computed 4-state profile by the MultiFoXS flexibility approach is presented in the inset. Goodness of the fit is expressed in χ^2^ (**b**) Ensemble of 4-state top best-scoring structures, superposed to the Ig domain 2 with the corresponding fractions.

However, EOM-produced models using separate Ig domains for fitting have some clashes between Ig domains and the dummy atoms of the linker. To overcome this problem, we prepared a model of Ig1–2 together with the linker (I-TASSER) and employed MultiFoXS server to explore the multiple conformations by conformational sampling with rapidly exploring random trees (RRT) search. Here, the linker residues were assigned as flexible. This significantly improved the fitting to the experimental data for 4-state model, showing lowered chi value (χ^2^ = 1.120) in comparison with the rigid body modelling (for comparison see Fig. 2c and Fig. 3a, inset). Radius of gyration distribution plot for different best scoring N-state models revealed a trimodal R_g_ peak distribution with maxima at 2.2, 2.6 and 2.8 nm (Fig. 3a), which is in excellent agreement with the data calculated by the EOM method. Graphical representation of the calculated multi-state model confirmed the presence of the different populations of tandem Ig domain arrangements (Fig. 3b). A similar distribution was observed for the semi-extended Ig domain pair 6–7 of filamin C^49^. Taken together, these results reveal that tandem Ig domains of myotilin display a certain degree of flexibility and exist in different relative orientations, most likely due to the flexible nature of the linker between the two domains.

#### Molecular modelling and dynamics

In order to confirm flexibility and dynamics of Ig domains of myotilin shown by SAXS measurements, we employed molecular dynamics simulations. First, we produced models of tandem Ig domains of myotilin using I-TASSER, with the Z-score as a selection criteria. Comparatively, best model generated in the Swiss-model repository showed almost identical structure with the RMSD value of 1.16 Å between 191 equivalent C_α_ atoms and almost coaxial orientation. For the molecular dynamics simulations the best model generated by I-TASSER was used.

**Figure 4:**
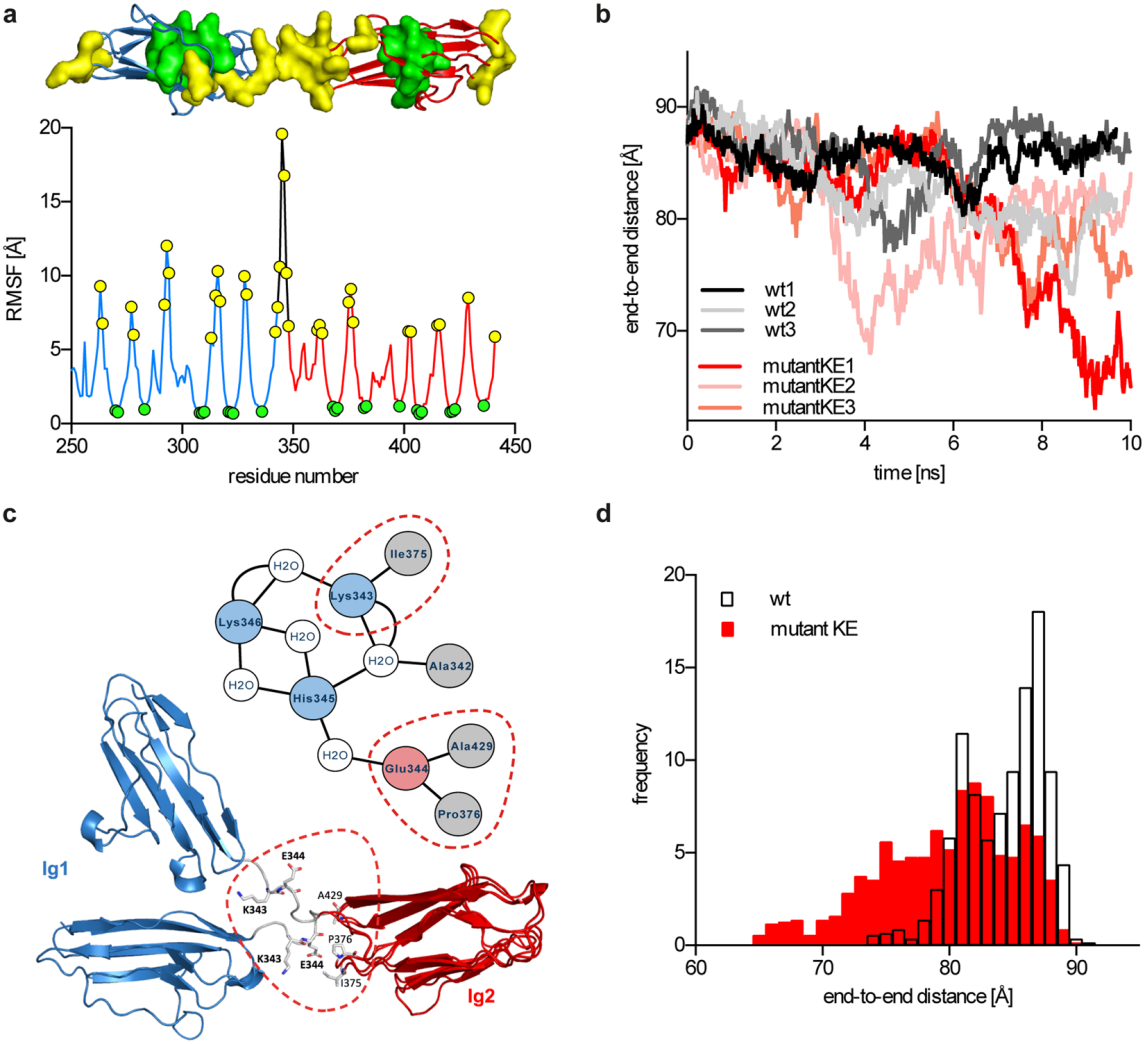
Molecular dynamics simulations. (**a**) RMSF plot from the coarse-grained simulation by the CABS-Flex server. Residues with the highest and lowest RMSF values are shown as yellow and green circles, respectively. Residues with the highest and lowest values are presented on the structure of MYOTIg1-2 with the same colouring scheme as in RMSF plot. (**b**) All atom, 10 ns simulations of the MYOTIg1-2 (wt) and KE->AA mutants. End-to-end distances through the course of simulations are shown, where Ig domains adopt compact conformation, followed by the reversible extension. (**c**) Presentation of the extended and compact forms of MYOTIg1-2, showing rotation of the highly conserved Glu344 around the main chain and its involvement in the torsion mechanism. Residues Ile375, Pro376 and Ala429 participate in the hydrogen bonding with the Glu344 and Lys343 and are marked with the red dotted circles. Intra-domain hydrogen bonding of linker residues, analysed through the course of simulation is shown in the inset. Negative charged amino acid residues are coloured red, positive blue, hydrophobic grey and water molecules white. (**d**) End-to-end distance distribution plot, where wt and mutant KE are showing different distribution.

Residues with highest degree of flexibility were predicted using a coarse-grained dynamics approach implemented in CABS-Flex server. Here, high root mean square fluctuation (RMSF) value is indicative of high residue flexibility. For several other proteins, results of such simulations have already been proved to be in good agreement with NMR measurements and all-atom MD simulations aimed to determine flexible regions^52^. The residue fluctuation profile of Ig1–2 domain pair of myotilin shows that highest flexibility is associated with the linker region between Ig1 and Ig2 while residues of the Ig domains’ hydrophobic core have far lower RMSF values and are hence less flexible (Fig. 4a). Fluctuations in N-to-C-terminus distance of Ig1–2 domain pair during the coarse-grained simulation indicate a transition from fully-extended (starting model) to a more compact domain arrangement.

Detailed analysis of fluctuations in domain arrangement was analysed using two 10 ns all-atom MD simulations. Here, the Ig1–2 domain pair moved from an extended to a more compact arrangement, followed again by a transition to regain a locally more extended form. In case of simulation 1, we observed almost complete regain to extended orientation (Fig. 4b), whereas simulation 2 showed significantly more compact domain orientation at the end of simulation. More extended orientation and limited flexibility in simulation 1 could originate from the observed formation of short α-helix of linker residues. These residues showed preference for the α-helical structure, as was corroborated by structure prediction with I-TASSER and PEP-FOLD3, showing helical structure of all produced models.

During simulation 2 the maximum relative rotation angle between the domains reached a value of 104° with the domain closure of 54.5 % as analysed by the DynDom server (here, values of 0 and 100 % correspond to fully extended and fully compact forms, respectively). Further analysis of the extended and compact conformations from the all-atom MD simulations revealed that the residue stretch ^344^EHKR^347^ represents a hinge, where the main chain bond rotation results in the domain torsion (Fig. 4c). This hinge region is also involved in the formation of the α-helical region observed during molecular dynamics simulations. Interestingly, the Glu residue within this stretch is also conserved in the linker regions of tandem domains of titin where it was suggested to be involved in intra- and inter-domain stabilisation, however no structural data is available to unequivocally confirm this hypothesis^33^. Our data demonstrate that the corresponding Glu residue of myotilin (Glu344), in concert with other hinge residues, plays an important role in hinge mechanism of myotilin, where its main dihedral ψ angle changes by 54° as measured by the DynDom server. Moreover, formation of intra-domain interactions via hydrogen bonds was observed between Lys343 and Ile375 and between Pro376/Ala429 and Glu344 (Fig. 4c). Remarkably, Pro376 and Ala429, located within the Ig2 domain, are part of the highly conserved motifs PxP and NxxG. Additional MD simulations were performed with the *in silico* mutated AKE motif to AAA, where we observed increased flexibility and less extended structure of the mutant, suggesting that the AKE motif is important for the stabilisation of the inter-domain orientation. Additionally, distribution of end-to-end distances differs for wt and mutant (Fig. 4d). Distribution of mutant is significantly broader (median = 80.2 Å, SD = 5.5 Å), compared to the wt (median = 84.6 Å, SD = 3.2 Å).

Therefore, our data clearly demonstrates a connection between conserved motifs and myotilin dynamics on a level of intra-domain contacts involving residues within the conserved motifs.

## Discussion

To date, great effort has been put in studies of mechanical and structural properties of sarcomeric proteins located within the I-band and Z-disk, particularly with regard to their single and poly-tandem Ig domain regions which are known to be able to adopt different relative orientations and in turn affect the flexibility and elasticity of their cognate proteins. In particular, the main focus of the research has been elastic I-band region of titin and inter-domain interactions of filamins. Results have shown essential impact of titin domain unfolding, stability and inter-domain mobility on mechanical properties directly correlated to muscle contraction and elasticity^53^. Studies of the titin N-terminal Z1Z2, located within the Z-disc^34^, have revealed important implications of inter-domain dynamics in unbound state for interaction with binding partner telethonin^35^. Also, compact domain-domain interactions of filamins^30, 31, 49, 54^, as well as inter-molecular interaction between Ig24 domains leading to dimerisation^55, 56^, were found to be functionally important. For example, relative orientation and inter-domain contacts of filamin Ig domains 16–24 (Rod 2 region) play an important role in ligand binding regulation by the β sheet augmentation mechanism^30^. Additional example of such multi-domain protein is myomesin, located at the M-band. Crystal structures of its poly-tandem Ig9–13 domains revealed structured, α-helical linkers between domains, which restrict domain flexibility. These domain-connecting linkers possess a spring-like mechanism – they unfold upon applied tension and again refold when tension is reduced^57, 58^.

Here, we have focused on a small family of Z-disc proteins, composed of myotilin, myopalladin and palladin. Myotilin is the smallest member of the family with the two Ig domains, compared to a modular proteins myopalladin and palladin, which possess up to five Ig domains. To date, only a limited structural and mechano-dynamical data exist for this family; while structures of single myotilin Ig1 (ref. 59) and Ig2 and palladin Ig3 (ref. 60) and Ig4 domains have been determined, no information about the tandem domain orientations and dynamics is available.

SAXS data, which allowed us to produce averaged low-resolution molecular envelope of tandem myotilin Ig domains, revealed »dumbbell« shape with an almost extended domain conformation. Flexibility analysis using dimensionless Kratky plot suggests a certain degree of conformational plasticity, which was confirmed using ensemble optimisation method (EOM) and MultiFoXS approaches. Use of these methods significantly improved fitting to the experimental data, compared to the rigid body modelling, and showed that myotilin Ig domains in solution can be described as a multi-conformation species that adopt various relative orientations resulting in a compact or more extended forms. However, these states are not equally populated since in the predominant form (50 %) the Ig domain pair adopts a semi-extended orientation. Interestingly, a preferential orientation has also been reported for the Z1Z2 domain pair of titin with implications in selective recruitment of the binding partner telethonin^34^. Since myotilin Ig domains present an important interaction site for prominent proteins of the sarcomere like F-actin and filamin C, Ig domain flexibility could be a prerequisite for is interaction capabilities in the unbound state. We propose that conformational plasticity of Ig domains is part of the binding partner recognition mechanism similarly as reported for Z1Z2 upon binding to telethonin^34^.

While previous studies showed that myotilin is able to form dimers via Ig domains^4, 8, 9^, we did not observe species with molecular weight corresponding to the mass of the dimer (calculated from I_0_ values), which indicates that the concentration effect described above was not a consequence of oligomerisation. Different construct design used in our and in previously reported studies suggests that regions outside of the Ig1–2 domain pair promote dimer formation and/or that the conditions used do not favour the formation of a stable dimer. For example, study of Salmikangas et. al showed that construct encompassing residues 229–441 is able to dimerise^8^. On the other hand, another study revealed that smallest fragment of myotilin with the ability to dimerise is 345–498 (ref. 9).

Molecular dynamics simulations using coarse-grained and all-atom approaches corroborated these findings and further showed that the transition of extended form to the more compact one is reversible. Specifically, we identified the highly conserved Glu344, located in the middle of the linker, as a central point of the torsion mechanism involving the rotation of dihedral angle ψ around the main chain. Presence of this conserved acidic residue within the linker sequence was already reported for I-band Ig domains, but its involvement in intra- or inter-domain interactions was not clearly shown^33^. In case of myotilin Ig domains, we noticed that during simulation Lys343 and Glu344 formed H-bonds with the Ile375 and Pro376/Ala429, respectively, within the N-terminal PxP and NxxG motifs on Ig2 domain, indicating impact of these conserved features on intra-domain stabilising interactions. Moreover, we observed that linker residues during simulation showed tendency to form α-helical structure, therefore restricting flexibility and consequently affecting Ig domain dynamics.

Molecular evolution analysis of the myotilin revealed that Ig domains are the most conserved part and present the only structured region of the molecule, consistent with the disorder prediction. When only Ig domains were analysed, mainly hydrophobic residues were conserved, along with the PPxf and NxxG motifs, whereas other solvent accessible residues showed high variability, implying that these residues co-evolved in respect to the binding partners. Linker residues between homologous Ig domains of the family were highly conserved and it can be speculated that they share similar mechanisms of intra-domain orientation changes in contrast to other Ig domain pairs in palladin and myopalladin, which have different linker length and amino acid composition.

Our molecular evolution analysis additionally allowed us to shed light on the molecular basis of myotilinopathies. Based on our results, we propose that aggregation through newly exposed hydrophobic patches plays an important role in pathological mechanism, probably in concert with the slower degradation and changed turnover as already indicated in previous studies^28^.

In conclusion, our results clearly show that Ig domain pair of myotilin is flexible, however with a general preference for a semi-extended inter-domain orientation with possible implications in binding partner recognition by analogy to other known examples of tandem Ig domain-containing proteins. Also, due to similar linker length and high sequence conservation, similar tandem Ig domain dynamics is expected for myopalladin and paladin, which is in line with their partially overlapping roles.

## Methods

### Cloning, protein expression and purification

DNA fragment encoding Ig1–2 domains of human myotilin (residues 250–444; Uniprot accession code Q9UBF9) was cloned into pETM-14 vector (EMBL) to create fusion construct with the plasmid-encoded N-terminal His_6_-tag and a HRV-3C protease cleavage site at the 5′ end (MYOTIg1–2). Correct assembly was verified by DNA sequencing (GATC Biotech). For protein expression *E. coli* strain BL31[DE3] was used. Briefly, cells were grown at 37 °C in LB media supplemented with ampicillin (100 µg/mL) to an OD_600_ of approximately 0.6. After cooling to 20 °C recombinant protein expression was induced by addition of IPTG to a final concentration of 0.5 mM. After 16 h cells were collected by centrifugation, resuspended in lysis buffer (20 mM HEPES, 500 mM NaCl, 10 mM imidazole, pH 7.4), and lysed by sonication. Soluble fraction was obtained by centrifugation (20 000 rpm, 4 °C) and applied onto HiTrap IMAC FF column (GE Healthcare). After washing (20 mM HEPES, 500 mM NaCl, 10 mM imidazole, 5 % glycerol, pH 7.4) the bound proteins were eluted by gradually raising imidazole concentration to 500 mM. His_6_-tag was removed by incubation of the pooled eluates containing MYOTIg1–2 with GST-tagged HRV-3C protease (prepared in-house; mass ratio MYOTIg1–2/HRV-3C 100:1) during overnight dialysis at 4 °C against the dialysis buffer (50 mM Tris, 150 mM NaCl, 1 mM DTT, pH 7.4). The His_6_-tag free MYOTIg1–2 was recovered as a flow-through after applying the cleavage mixture onto HiTrap IMAC FF and GSTrap FF columns to remove uncleaved MYOTIg1–2 and HRV-3C protease, respectively. Final purification step was size-exclusion chromatography on a Superdex 75 10/300 column (GE Healthcare) equilibrated in buffer 20 mM HEPES, 150 mM NaCl, 5 % glycerol, 1 mM DTT, pH 7.4. The purity of the final samples was assessed by SDS-PAGE (Supplementary Fig. S3).

### Evolutionary analysis

For evolutionary analysis, two sets of data were prepared: (1) complete myotilin sequences (Supplementary Table S1) and (2) sequences of Ig domains of myopalladin and palladin (Supplementary Table S2) from Vertebrates subphyla. Sequences were retrieved from the NCBI nr database via Protein Blast and Ensembl genome browser using either myotilin (Q9UBF9) or Ig domain sequences of palladin (Q8WX93) and myopalladin (Q86TC9). Following manual data inspection and filtering, the sequences were aligned and phylogenetic trees were constructed using maximum likelihood based method implemented in the MEGA 7 software^61^. Input parameters for constructing phylogenetic trees were: LG substitutional model, Bio-NJ as initial tree, Nearest-Neighbour-Interchange (NNI) as tree inference heuristic method and branch support calculated with 100 bootstrap replicates. Calculated trees were graphically edited using FigTree software (http://tree.bio.ed.ac.uk/software/figtree/). Conservation scores for each myotilin residue in the multiple sequence alignment (MSA) were calculated using the Scorecons server^62^. Disorder tendency prediction was performed using the GeneSilico MetaDisorder server^63^.

ConSurf-DB^64^ was used for presentation of evolutionary conservation profiles of myotilin Ig domains. Pre-calculated MSA for Ig1 consisted of 301 homologues and 152 homologues for Ig2. Calculated conservation rates were projected onto both Ig structures and coloured according to the used colouring scheme.

### Small-angle X-ray scattering (SAXS)

SAXS data for purified construct MYOTIg1–2 was collected at ESRF beamline BM29 BioSaxs (Grenoble, France) equipped with the Pilatus 1M detector. Samples were measured at concentrations of 3.67, 7.00 and 14.12 mg/mL in buffer 20 mM Na^+^-HEPES, 150 mM, NaCl, 5 % glycerol, 1 mM DTT, pH 7.4, in two independent measurements. The scattering intensity (I) was measured in the range from 0.03 < s < 5.0 Å, where s = 4π sinθ/λ and λ = 1 Å, at the distance between sample and detector of 2.867 m. Background scattering was subtracted, data reduced, normalised according to the measured concentration and extrapolated to infinite dilution using the two lowest measured concentrations using PRIMUS^65^ module of the ATSAS software package^66^. Forward scattering (I_0_) and radius of gyration (R_g_) were obtained by fitting the linear Guinier region of the data. Pair distribution function P(r) with the corresponding maximum particle size parameter (D_max_) was determined using GNOM program^67^. The dimensionless Kratky plots were generated by the use of data from SASBDB^50^ for BSA (accession code: SASDBT4), filamin A Ig20-Ig21 (accession code: SASDAH8), filamin A Ig20-Ig21+migfilin (accession code: SASDAG8) and filamin A Ig22-Ig23 (accession code: SASDAU3).

For reconstruction of theoretical molecular envelope *ab initio* modelling was performed 20 times using the program DAMMIF^68^, where scattering from the calculated envelopes were fitted against the experimental scattering and evaluated by the chi values. The most typical envelope was selected by comparing the normalised spatial discrepancy (NSD) values between pairs of envelopes (Supplementary Table S3) and later averaged by DAMAVER set of programs^69^. In rigid body modelling, high resolution structures of myotilin Ig1 (PDB accession code: 2KDG) and Ig2 (PDB accession code: 2KKQ) were used to fit SAXS scattering data using program CORAL^66^. Linker residues were designated as dummy atoms. *Ab initio* calculated envelope was superposed to the rigid body model using SUPCOMB program^70^.

Flexibility analysis was performed using EOM2.1 program^71^ with enforced P1 symmetry while the linker between Ig domains was again designated as dummy atoms. Conformations consistent with scattering data were selected from the pool of 50 000 models using a genetic algorithm. The flexibility analysis was independently repeated three times; all runs gave comparable results. For comparative analysis MultiFoXS server^72^ was used where I-TASSER structure was used as the input model; residues Leu341, Ala342, Lys343, Glu344, His345, Lys346, Arg347 were designated as flexible residues. More than 10 000 conformations were calculated and scored with the rapidly exploring random trees (RRT) search to extract only those conformations, which adequately describe observed system. All structure figures were prepared using PyMOL (The PyMOL Molecular Graphics System, version 1.3, Schrödinger, LLC).

### Modelling, coarse-grained and all-atom molecular dynamics

Models of tandem Ig domains were calculated using I-TASSER^73^ and the best model was selected on the basis of its Z-score. This model was then used to analyse structure fluctuations as well as in molecular dynamics simulations. Same model was used for the *in silico* mutagenesis, which was performed in PyMOL, to obtain model with the mutated AKE motif to AAA.

Fluctuations of relative orientation between the tandem Ig domains were predicted using CABS-Flex server which implements structure coarse-graining and Monte Carlo dynamics sampling scheme^74^. RMSF values of the 2000 frames were analysed in terms of distance between Ig domains (defined as distance from C_α_ of N-terminal to C_α_ of C-terminal amino acid residue) *vs*. number of frames.

All-atom molecular dynamics (MD) simulations were performed using NAMD 2.1 program suite^75^ with CHARMM-27 force-field. Initial structure was solvated and neutralised (by adding Na^+^ or Cl^−^ ions) and the topology was generated using psfgen. Equilibration was done at 300 K using constant temperature Langevin dynamics, constant pressure via Noose-Hoover Langevin piston and Particle Mesh Edward for full-system periodic electrostatics. Energy minimisation was done for 500 steps of conjugate gradients. Time-step was set to 2 fs. The length of the final production run was 10 ns. MD trajectories were analysed using VMD^76^ where end-to-end distances were measured. Further trajectory analysis was done using the DynDom server^77^ to reveal hinge residues which could participate in the torsion mechanism of conformational switching and to calculate rotation angle of inter-domain bending. Inter- and intra-domain interactions via hydrogen bonding were analysed and graphically presented using Cytoscape^78^ coupled with UCSF Chimera^79^ and structureViz^80^ programs.

## Electronic supplementary material

Supplementary Information

Supplementary Information
