# Supplementary material for: Conformational plasticity and evolutionary analysis of the myotilin tandem Ig domains
Source: Sci Rep. 2017 Jun 21;7:3993. doi: 10.1038/s41598-017-03323-6 (PMC5479843; doi:10.1038/s41598-017-03323-6)
Supplement: Supplementary file 2 — Supplementary Information [file 41598_2017_3323_MOESM2_ESM.pdf]

# Conformational plasticity and evolutionary analysis of the myotilin tandem Ig domains

Vid Puž, Miha Pavšič, Brigita Lenarčič* & Kristina Djinović-Carugo*

## Supplementary Figures

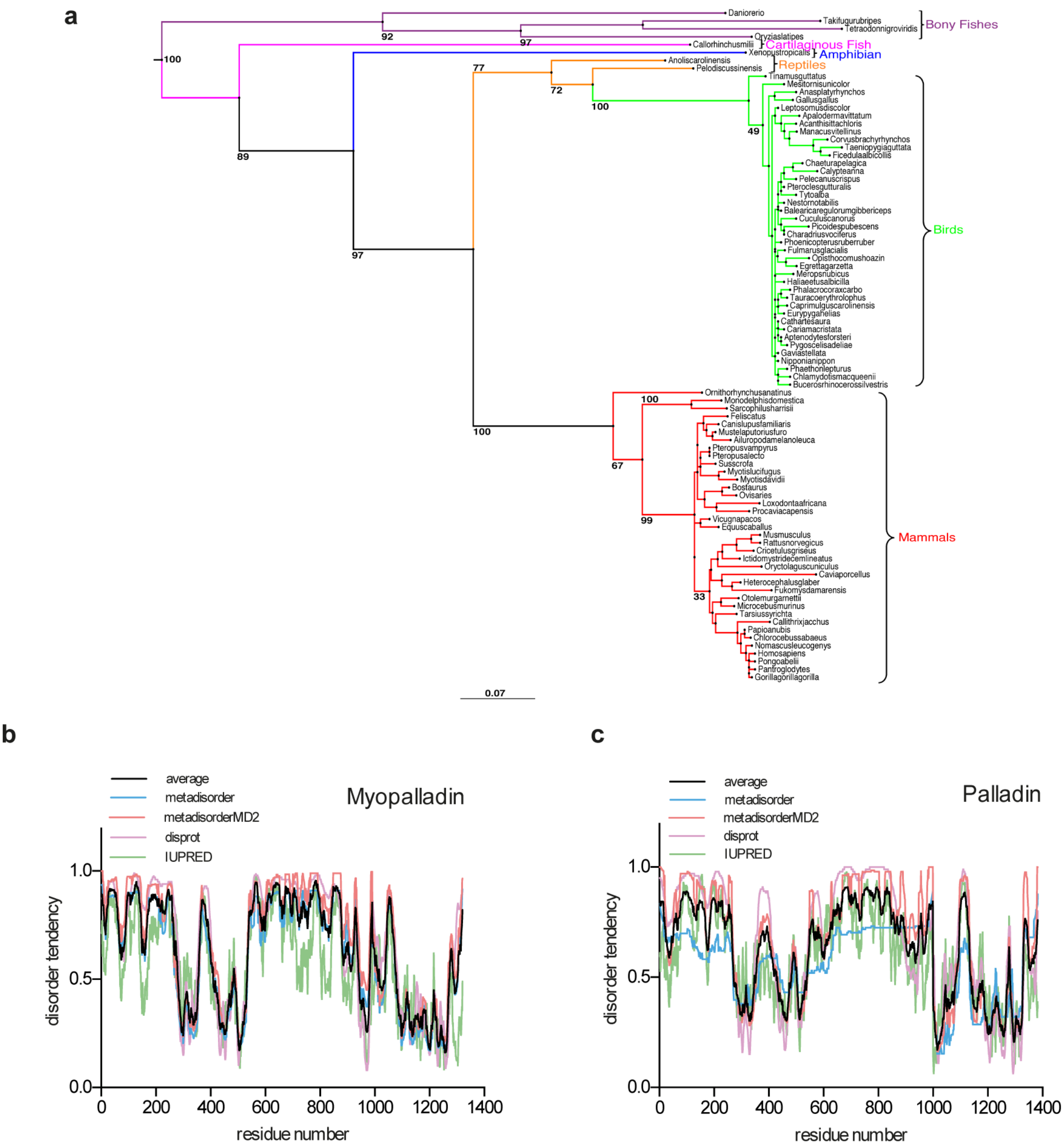


**Supplementary Figure S1. Phylogenetic trees and disorder tendency plots for evolutionary analysis of myotilin/myopalladin/palladin family.**

(**a**) Bio-NJ phylogenetic tree of myotilin, calculated from the dataset of 85 homologous myotilin sequences from Chondrichthyes to Mammalia classes, showing expected intra-species relationships with the strong branch support. Disorder tendency plots, calculated for myopalladin (**b**) and paladin (**c**), showing unstructured regions between the Ig domains.

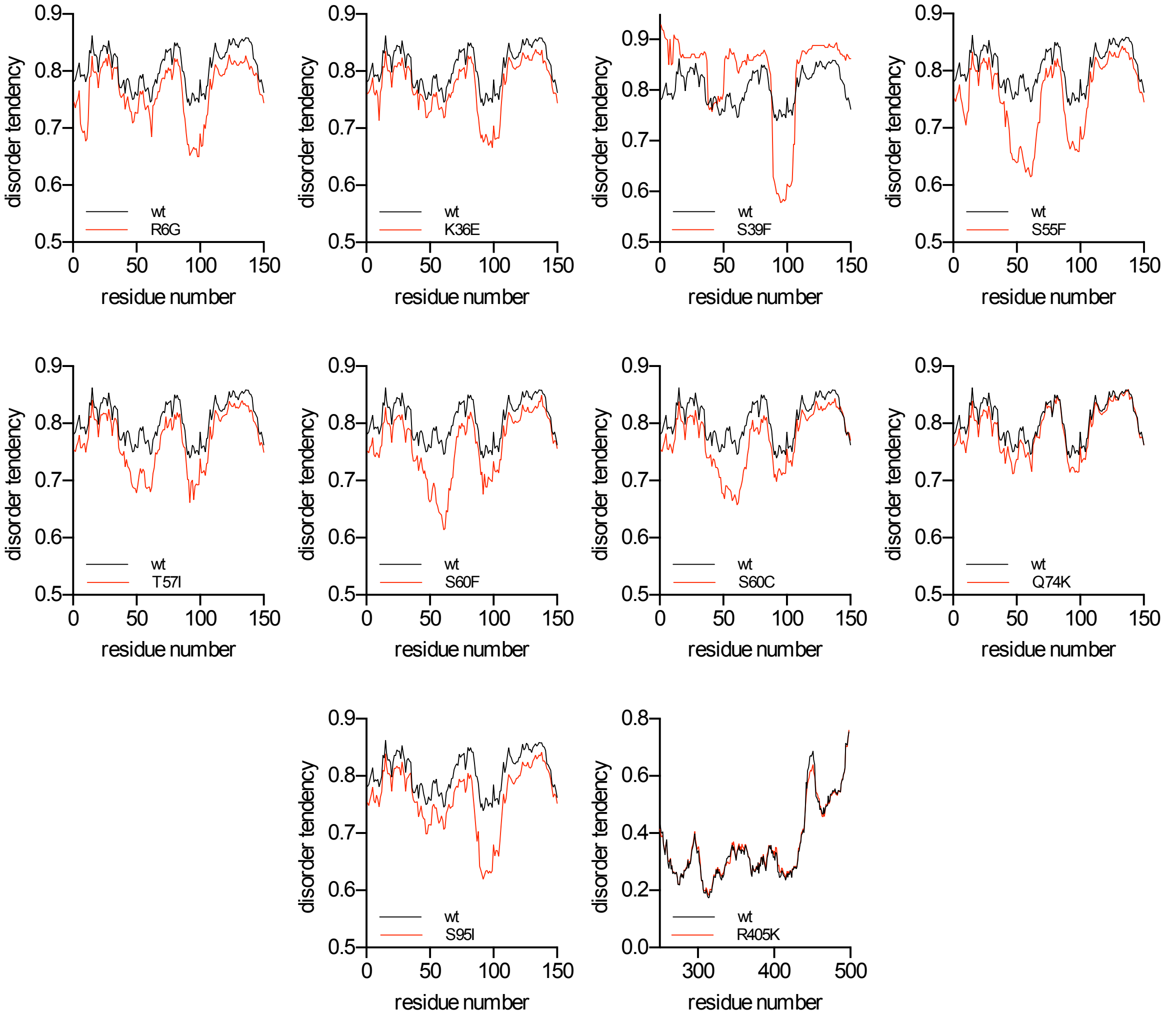


**Supplementary Figure S2. Analysis of disorder for wt and mutants of myotilin.**
Disorder tendency plots calculated for wt and disease-causing mutants of myotilin. Mutants are showing lower tendency scores, compared to the wt.

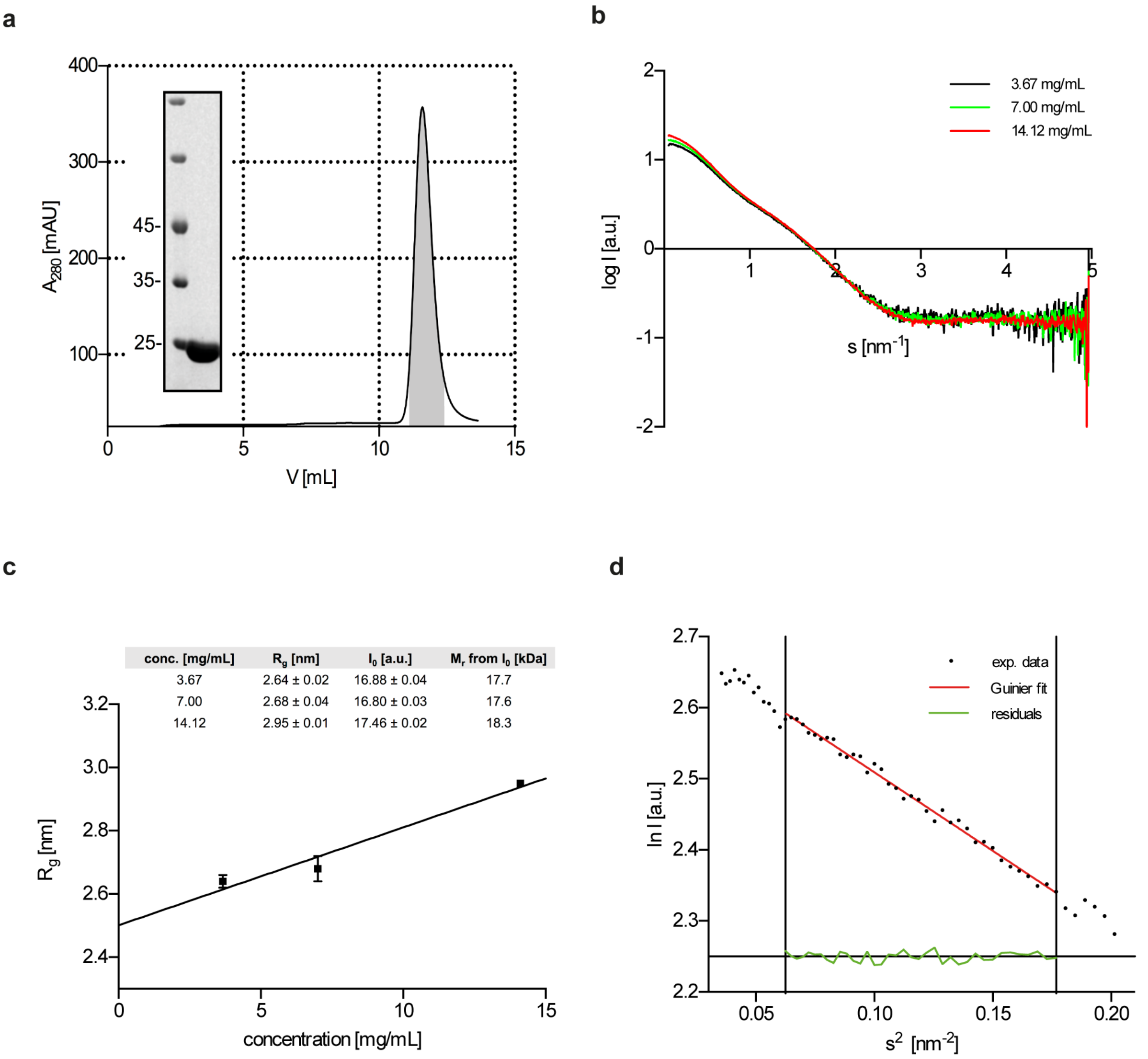


**Supplementary Figure S3. Sample purity and concentration effect check for SAXS.**
(**a**) Size-exclusion chromatography of the MYOTIg1-2, representing high sample purity, eluting as a monomeric species. SDS-PAGE analysis of the pooled fractions from chromatography, showing high purity of the sample (up to 95 %). (**b**) Scattering profiles (logarithmic scattering I *vs*. momentum transfer s) of MYOTIg1-2 at three different concentrations after background subtraction and concentration normalization (**c**) Concentration effect, observed in the $R_g$ plotted *vs*. concentration of the sample. Calculated masses, based on the $I_0$ values do not show any preference for the dimer formation. (**d**) Guinier plot showing linearity in the low s region. Fit of the linear region is shown in red, with the residuals shown in green.

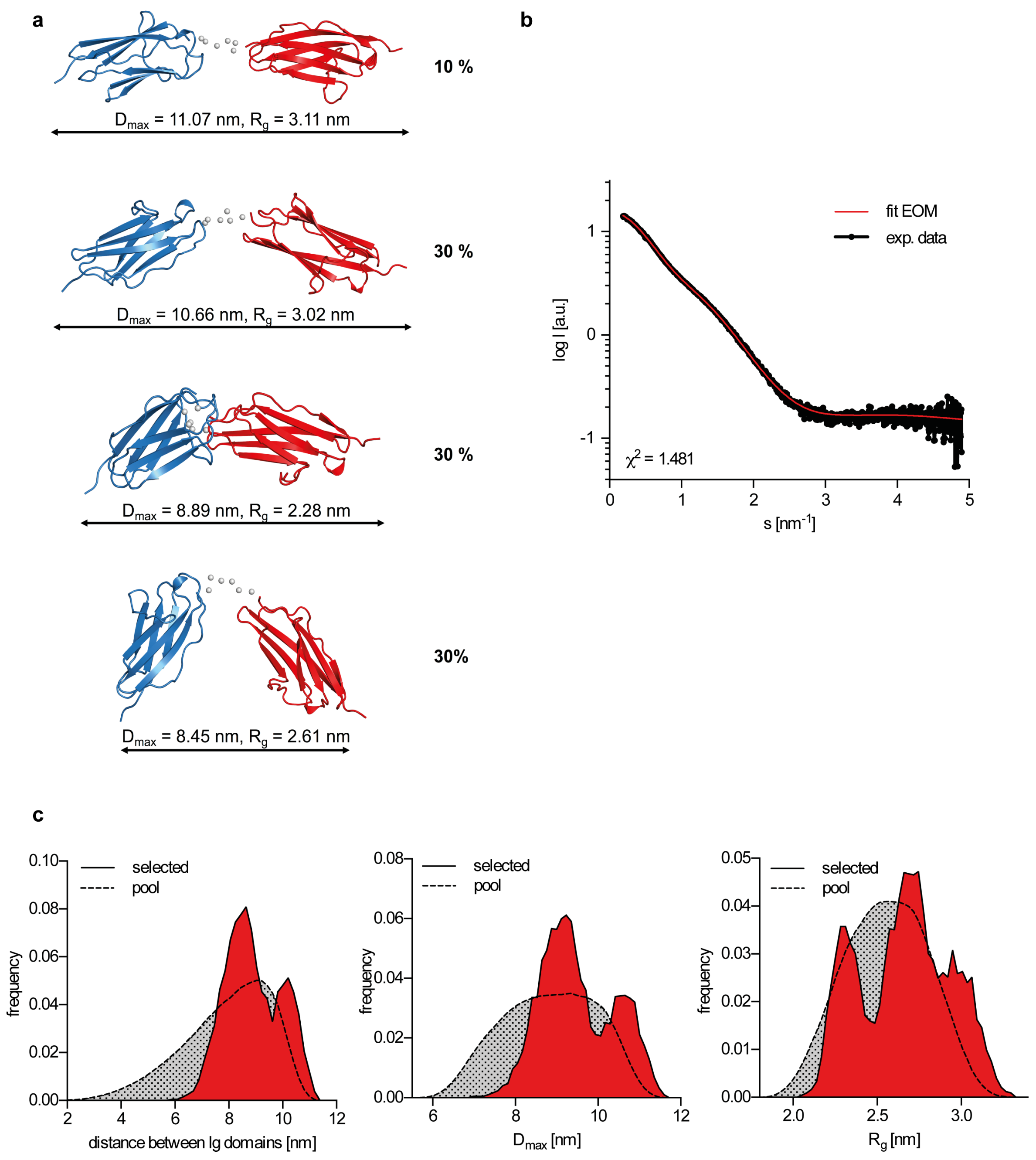


**Supplementary Figure S4. EOM analysis of MYOTIg1-2.**

(**a**) Four EOM-selected structures are shown with the corresponding fractions and parameters $D_{max}$ and $R_g$. (**b**) Fit of the experimental data with the EOM flexibility approach, with the goodness of the fit expressed in $\chi^2$. (**c**) Distribution plots of $R_g$, $D_{max}$ and end-to-end distances as selected from the pool of structures, represented by the dotted line. Trimodal profile can be observed for $R_g$, whereas $D_{max}$ and end-to-end distance plots show bimodal distribution.

**Supplementary Table S1. Accession codes of the myotilin sequences used for construction of Bio-NJ tree.**

| Species name | Name in phylogenetic tree | Accession code | Number of aa |
|---|---|---|---|
| Homo sapiens | Homosapiens | NP_006781.1 | 498 |
| Heterocephalus glaber | Heterocephalusglaber | EHB03270.1 | 496 |
| Fukomys damarensis | Fukomysdamarensis | KFO19208.1 | 497 |
| Pteropus alecto | Pteropusalecto | ELK03058.1 | 499 |
| Cricetulus griseus | Cricetulusgriseus | ERE81500.1 | 496 |
| Myotis davidii | Myotisdavidii | ELK23735.1 | 743 |
| Tinamus guttatus | Tinamusguttatus | KGL84392.1 | 502 |
| Manacus vitellinus | Manacusvitellinus | KFW78566.1 | 511 |
| Phalacrocorax carbo | Phalacrocoraxcarbo | KFW88146.1 | 508 |
| Nestor notabilis | Nestornotabilis | KFQ42305.1 | 512 |
| Cathartes aura | Cathartesaura | KFP49602.1 | 512 |
| Phoenicopterus ruber ruber | Phoenicopterusruberruber | KFQ85431.1 | 512 |
| Merops nubicus | Meropsnubicus | KFQ19336.1 | 512 |
| Haliaeetus albicilla | Haliaeetusalbicilla | KFQ11561.1 | 512 |
| Leptosomus discolor | Leptosomusdiscolor | KFQ02000.1 | 502 |
| Cariama cristata | Cariamacristata | KFP68757.1 | 512 |
| Balearica regulorum gibbericeps | Balearicaregulorumgibbericeps | KFO11505.1 | 512 |
| Gavia stellata | Gaviastellata | KFV53598.1 | 512 |
| Fulmarus glacialis | Fulmarusglacialis | KFV93003.1 | 512 |
| Chaetura pelagica | Chaeturapelagica | KFU83907.1 | 512 |
| Tauraco erythrolophus | Tauracoerythrolophus | KFV09571.1 | 512 |
| Caprimulgus carolinensis | Caprimulguscarolinensis | KFZ54626.1 | 512 |
| Phaethon lepturus | Phaethonlepturus | KFQ71710.1 | 512 |
| Opisthocomus hoazin | Opisthocomushoazin | KFR06853.1 | 512 |
| Eurypyga helias | Eurypygahelias | KFV94577.1 | 510 |
| Nipponia nippon | Nipponianippon | KFR04404.1 | 512 |
| Corvus brachyrhynchos | Corvusbrachyrhynchos | KFO58282.1 | 512 |
| Pterocles gutturalis | Pteroclesgutturalis | KFU96906.1 | 511 |
| Picoides pubescens | Picoidespubescens | KFV61279.1 | 512 |
| Tyto alba | Tytoalba | KFV47473.1 | 512 |
| Chlamydotis macqueenii | Chlamydotismacqueenii | KFP36037.1 | 512 |
| Egretta garzetta | Egrettagarzetta | KFP17987.1 | 512 |
| Acanthisitta chloris | Acanthisittachloris | KFP83155.1 | 517 |
| Charadrius vociferus | Charadriusvociferus | KGL87007.1 | 512 |
| Buceros rhinoceros silvestris | Bucerosrhinocerossilvestris | KFO87581.1 | 512 |
| Aptenodytes forsteri | Aptenodytesforsteri | KFM12062.1 | 511 |
| Mesitornis unicolor | Mesitornisunicolor | KFQ37832.1 | 507 |
| Calypte anna | Calypteanna | KFO97438.1 | 512 |
| Pygoscelis adeliae | Pygoscelisadeliae | KFW66160.1 | 511 |
| Apaloderma vittatum | Apalodermavittatum | KFP88858.1 | 502 |
| Pelecanus crispus | Pelecanuscrispus | KFQ48994.1 | 512 |
| Cuculus canorus | Cuculuscanorus | KFO69919.1 | 513 |

| | | | |
|---|---|---|---|
| Mus musculus | Musmusculus | NP_001028793.1 | 496 |
| Mustela putorius furo | Mustelaputoriusfuro | XP_004744962.1 | 498 |
| Bos taurus | Bostaurus | NP_001068803.1 | 499 |
| Sus scrofa | Susscrofa | NP_001093411.1 | 499 |
| Pongo abelii | Pongoabelii | XP_002815965.1 | 490 |
| Anolis carolinensis | Anoliscarolinensis | ENSACAT00000017158.3 | 501 |
| Callithrix jacchus | Callithrixjacchus | ENSCJAT00000011939.1 | 498 |
| Pelodiscus sinensis | Pelodiscussinensis | ENSPSIT00000016730.1 | 518 |
| Ictidomys tridecemlineatus | Ictidomystridecemlineatus | ENSSTOG00000013748.2 | 496 |
| Canis lupus familiaris | Canislupusfamiliaris | ENSCAFT00000001749.3 | 499 |
| Myotis lucifugus | Myotislucifugus | ENSMLUT00000010168.2 | 497 |
| Oryctolagus cuniculus | Oryctolaguscuniculus | ENSOCUT00000014413.3 | 499 |
| Papio anubis | Papioanubis | ENSPANT00000027253.1 | 498 |
| Ovis aries | Ovisaries | ENSOART00000016880.1 | 499 |
| Sarcophilus harrisii | Sarcophilusharrisii | ENSSHATG00000009931.1 | 499 |
| Ailuropoda melanoleuca | Ailuropodamelanoleuca | ENSAMET00000017951.1 | 497 |
| Cavia porcellus | Caviaporcellus | ENSCPOT00000001672.2 | 502 |
| Nomascus leucogenys | Nomascusleucogenys | ENSNLET00000010926.1 | 498 |
| Xenopus tropicalis | Xenopustropicalis | ENSXETT00000050537.2 | 498 |
| Rattus norvegicus | Rattusnorvegicus | ENSRNOT00000070817.2 | 496 |
| Ficedula albicollis | Ficedulaalbicollis | ENSFALT00000007984.1 | 509 |
| Equus caballus | Equuscaballus | ENSECAT00000022238.1 | 498 |
| Otolemur garnettii | Otolemurgarnettii | ENSOGAT00000016525.2 | 498 |
| Ornithorhynchus anatinus | Ornithorhynchusanatinus | ENSOANT00000014777.2 | 504 |
| Gorilla gorilla | Gorillagorilla | ENSGGOT00000014566.2 | 498 |
| Pan troglodytes | Pantroglodytes | ENSPTRT00000031968.5 | 498 |
| Callorhinchus milii | Callorhinchus milii | SINCAMT00000008039 | 521 |
| Monodelphis domestica | Monodelphisdomestica | ENSMODT00000015642.2 | 499 |
| Anas platyrhynchos | Anasplatyrhynchos | ENSAPLT00000013384.1 | 510 |
| Oryzias latipes | Oryziaslatipes | ENSORLT00000011256.1 | 445 |
| Tetraodon nigroviridis | Tetraodonnigroviridis | ENSTNIT00000013214.1 | 418 |
| Takifugu rubripes | Takifugurubripes | ENSTRUT00000016983.1 | 441 |
| Loxodonta africana | Loxodontaafricana | ENSLAFT00000026381.1 | 499 |
| Vicugna pacos | Vicugnapacos | ENSVPAT00000006316.1 | 499 |
| Felis catus | Feliscatus | ENSFCAT00000015551.3 | 499 |
| Gallus gallus | Gallusgallus | ENSGALT00000056777.1 | 513 |
| Procavia capensis | Procaviacapensis | ENSPCAT00000003695.1 | 499 |
| Pteropus vampyrus | Pteropusvampyrus | ENSPVAT00000002236.1 | 499 |
| Microcebus murinus | Microcebusmurinus | ENSMICT00000034389.1 | 491 |
| Tarsius syrichta | Tarsiussyrichta | ENSTSYT00000002660.1 | 499 |
| Chlorocebus sabaeus | Chlorocebussabaeus | ENSCSAT00000011412.1 | 498 |
| Taeniopygia guttata | Taeniopygiaguttata | ENSTGUT00000001239.1 | 497 |
| Danio rerio | Daniorerio | ENSDART000000115101.3 | 561 |

**Supplementary Table S3. List of the $\chi^2$ values for each DAMMIF-produced model and NSD values of models, calculated by the program damsel.**

| Model number | $\chi^2$ | NSD |
|---|---|---|
| 1 | 1.515 | 0.583 |
| 2 | 1.510 | 0.583 |
| 3 | 1.507 | 0.572 |
| 4 | 1.516 | 0.582 |
| 5 | 1.511 | 0.576 |
| 6 | 1.513 | 0.586 |
| 7 | 1.509 | 0.606 |
| 8 | 1.506 | 0.570 |
| 9 | 1.511 | 0.566 |
| 10 | 1.510 | 0.583 |
| 11 | 1.509 | 0.544 |
| 12 | 1.518 | 0.631 |
| 13 | 1.512 | 0.584 |
| 14 | 1.518 | 0.556 |
| 15 | 1.505 | 0.574 |
| 16 | 1.506 | 0.568 |
| 17 | 1.521 | 0.567 |
| 18 | 1.513 | 0.577 |
| 19 | 1.507 | 0.565 |
| 20 | 1.510 | 0.580 |

**Supplementary Data S1. Sequence alignment of the myotilin sequences of Vertebrates subphyla ranging from Chondrichthyes to Mammalia classes.**
